# When Measurements Meet Blockchain: On Behalf of an Inter-NMI Network †

**DOI:** 10.3390/s21051564

**Published:** 2021-02-24

**Authors:** Mahbuba Moni, Wilson Melo, Daniel Peters, Raphael Machado

**Affiliations:** 1 Department 8.5 Metrological Information Technology, Physikalisch-Technische Bundesanstalt (PTB), Abbestrasse 2-12, 10587 Berlin, Germany; daniel.peters@ptb.de; 2 Division of Metrology in Information Technologies and Telecommunications, Brazilian National Institute of Metrology, Quality, and Technology (Inmetro), Av. Nossa Sra. Das Graças 50, Duque de Caxias, Rio de Janeiro 25250-020, Brazil; wsjunior@inmetro.gov.br (W.M.J.); rcmachado@inmetro.gov.br (R.M.)

**Keywords:** blockchain, metrology digitalization, PKI, smart meter

## Abstract

The growing demand for solutions related to measurement (e.g., digital sensors, smart meters, distributed measuring systems) imposes several concerns about information and process reliability. In this context, blockchain can play a crucial role as a platform to implement applications and activities in the context of legal metrology. In most countries, the National Metrology Institutes (NMIs) are responsible for promoting these initiatives. Thus, in this paper, we present a functional architecture to integrate NMIs in a collaborative blockchain network. We discuss the main aspects and features that an inter-NMI blockchain network must deliver. Furthermore, we implement our proposal using the Hyperledger Fabric platform. We connect peers from Physikalisch-Technische Bundesanstalt (PTB) (German NMI) and the National Institute of Metrology, Quality, and Technology (Inmetro) (Brazilian NMI) in a useful application that consists of a blockchain-based public-key infrastructure to identify and authenticate smart meters. Our preliminary results demonstrate that the proposed architecture meets the main requirements imposed by applications involving measurements. Furthermore, it opens the opportunity to integrate NMIs from other countries into the project, constituting an important global initiative in the metrology field.

## 1. Introduction

In the modern world, the increasing digitalization of processes involving measurement introduces a significant challenge: ensuring the data’s and procedures’ reliability [1,2]. This challenge is especially relevant when one considers that in Europe alone, measuring instruments are responsible for an annual turnover of more than 500 billion Euros [3]. Currently, measuring instruments are devices that present high integration and connectivity with different technologies. One can mention smart meters (which constitute examples of IoT devices), distributed measuring systems (which usually involve sensor networks), and other smart components with complex software features [2,4,5,6]. Although these novel functionalities enhance efficiency and reduce costs, they also increase the attack surface on these systems and devices, introducing vulnerabilities and flaws. Thus, measurement applications demand mechanisms that attest to the reliability of any measuring instrument involved in the process and the integrity and authenticity of any provided information.

In this context, Legal Metrology (LM) plays a crucial role by providing confidence in the measurement of physical quantities [3,7]. LM depends on activities that aim to assure measuring instruments’ correct behavior in different applications. For example, it is responsible for promoting tests to assess instrument models and inspect these instruments in operational conditions. However, such trust has an intrinsic cost. LM activities need to deal with a significant amount of measuring instruments built from different technologies [8]. Consequently, processes such as model appraisal become excessively expensive for manufacturers and society. Furthermore, the inspection of many instruments distributed in large geographic areas constitutes a challenge of considerable proportions in terms of logistics and technical expertise [9,10].

We support the idea that LM also must take advantage of the digitalization of the activities [1,2,11]. LM needs to incorporate new technologies that make it easier to gather and share information about measuring processes’ verification and control. In this scope, a remarkable project is the European Metrology Cloud [1], a long-term project proposing the integration among different LM activities in a secure cloud computing architecture. The integrated processes include managing legal activities, measurement storage, monitoring systems, and logging of legally relevant activities. In Brazil, information technologies and management systems also have been applied to LM successfully, increasing the efficiency of processes regarding all levels of control [11].

Among all the technologies that can assist LM’s digitalization, blockchain emerges as a promising trend [12]. One can regard blockchain as a distributed append-only data structure (designated as a ledger), the integrity of which comes from the consensus among a set of network peers [13]. A blockchain can significantly reduce the costs associated with some LM activities, providing sophisticated mechanisms to ensure the integrity and authenticity of information and processes managed by the network [14]. In the last two years, different works have proposed blockchain-based solutions to classical scenarios related to LM [9,10,15,16,17]. The majority comes from joint efforts between the Physikalisch-Technische Bundesanstalt (PTB) and the Brazilian National Institute of Metrology, Quality, and Technology (Inmetro) (PTB and Inmetro are the National Metrology Institutes (NMIs) in Germany and Brazil, respectively.) However, all these mechanisms depend on a metrology blockchain network’s existence to become effective and practical solutions.

In this work, we propose and implement an inter-NMI blockchain network. This network constitutes the basis for developing sophisticated blockchain-based applications that employ smart contracts to establish digital processes in the LM context. This initiative allows cooperation among several NMIs. Each NMI can deploy solutions of interest to its respective country while supporting other NMIs in different scenarios involving measurement. The present paper is an extended version of one of our previous works [17] where we implemented a blockchain-based public-key infrastructure for securing smart meters. Now, we provide more details about this application by describing how an inter-NMI blockchain network can improve it. Our main new contributions are the following:

- We discuss the relevance of a permanent inter-NMI blockchain network. We describe how this initiative requires each country member’s commitment and how its availability can be worthwhile to them.
- We propose a blockchain network architecture based on a distributed and decentralized model. We explain why this model is the most indicated for the LM scope and foresee the technologies necessary to implement it.
- We implement our inter-NMI blockchain network using the Hyperledger Fabric platform. The implementation integrates servers from PTB and Inmetro in two different managing modes: the direct connection among peers and swarm-based orchestration. We discuss each modes and give directions about managing our inter-NMI network’s eventual growth.

## 2. Background

### 2.1. Legal Metrology and Digitalization

Legal Metrology (LM) is responsible for providing trust in physical quantities’ measurements in relations that involve consumption, security, life protection, and environmental preservation [3]. To do that, LM introduces control activities that assure the reliability of measuring instruments in different applications. The literature references these activities as type approval and metrological supervision (which includes marketing and field surveillance) [7]. They introduce practices and mechanisms that ensure the integrity of measuring instruments’ legally relevant chain. The term Legally Relevant (LR) refers to any part of a measuring instrument that can influence the generation and manipulation of sensitive information (i.e., measurements, sensors data, and digital evidence). In a measuring instrument, LR components exchange information and control actions logically, creating something like a dependency chain. For this reason, the protection of the LR chain is critical to ensure the reliability of any measuring instrument.

In all countries, the efficiency of activities related to LM constitutes a growing challenge. Mainly, software controlled measuring instruments (as is the case of smart meters) include new technologies that present higher complexity and introduce several security concerns. As an example of these concerns, one can mention LR chain integrity, the protection of sensitive information, and the availability of connected meters. These challenges assume more significant proportions in developing countries due to the dissemination of smart meters in complex scenarios and the high incidence of fraud in measurements [9,10,18].

In all these aspects, digitalization seems to be the best alternative to improve the activities related to LM [1,2,11]. A promising approach as an upcoming platform for legal metrology is the European Metrology Cloud (EMC), a coordinated European digital quality infrastructure for innovative products and services [1]. In general, the Metrology Cloud can be regarded as a distributed network because every stakeholder manages his/her own peer/server to participate in the network and keeps the data locally safe. These stakeholders are manufacturers, notified bodies, market surveillance authorities, and users of measuring devices. Hence, the platform needs authorization mechanisms to manage these stakeholders’ rights in different parts of the EMC.

### 2.2. Blockchain and Measurement Applications

Blockchain is an emerging technology that has caught stakeholders’ attention in different knowledge areas [19]. LM is also one of these areas, with proposals involving the measurements’ audit, information security and integrity, software protection, metrological surveillance, and distributed computing for measuring instruments [9,10,14,15,16,17].

One can define a blockchain as a distributed append-only data structure (designated as a ledger), which is replicated and shared among a set of network peers [19,20]. Blockchain ensures integrity and availability by consensus among peers [20]. This mechanism prevents any modification of the chain, enforcing an agreement about any new block in the ledger. A blockchain can store virtually any digital asset, from data to self-executing scripts, whereby the latter are called smart contracts. This enhances blockchain’s ability from being just a reliable data storage solution, to being a complete distributed platform for a proper automated workflow [13], in which smart contracts are executed by every assigned network peer that has permission, in an independent and automatic manner.

Blockchain technology’s features make it a great candidate to manage applications in scenarios like the EMC [12]. These applications demand that individual stakeholders agree about the correct state of information and processes independently. Features such as integrity assurance and smart contracts’ enforcement can be handy in improving different LM activities. Usually, LM faces different demands in different countries. For instance, the privacy and protection of sensitive information are among the most critical concerns for LM in the European Union. In turn, the reliability of measuring instruments and fraud prevention are the main drivers of LM in developing countries. In both scenarios, blockchains can contribute with different solutions. Another aspect is the management of activities involving type approval and surveillance. Mostly, these activities demand interaction among different parts, for instance measuring instruments’ manufacturers and users, vendors, consumers, notified bodies, and governments. These diverse stakeholders could reduce the costs and their processes’ complexity by integrating data and actions within a blockchain.

Recent works have proposed blockchain applications in different cases related to LM. Peters et al. [14] were the first to describe a set of applications in the context of LM that can explore blockchain’s properties. Melo et al. [5] described how to implement distributed measuring systems using smart contracts to execute LR software. The works of Peters et al. [15] and Yurchenko et al. [16] discussed how one can improve privacy in blockchain by using homomorphic and functional encryption in smart contracts. Finally, Melo et al. [10] presented a distributed and decentralized framework to implement fuel dispensers’ field surveillance using smart contracts. All these works describe ideas about implementing blockchain-based applications that involve LM. However, they lack how to create and manage a real blockchain network to support practical scenarios involving these solutions.

Upon creating a blockchain network to address LM demands, one needs to consider an architecture that matches these applications’ elementary requirements [12,21]. Broadly speaking, LM activities and applications demand the participation of stakeholders with distinct interests. In many cases, these interests can be conflicting. For instance, in the trading of measured goods, vendors usually expect a measuring instrument to work with the maximum admissible measuring error (i.e., more profitability). In contrast, consumers expect the opposite (i.e., lower price). Intermediary entities (e.g., notified bodies) act as mediators to ensure a fair trade. Besides, LM activities and applications also deal with sensitive information (e.g., energy measurements can expose consumers’ personal habits). Thus, a blockchain architecture in the LM context needs to promote the harmonic interaction among the parts while protecting information from undue access.

The literature usually classifies blockchain platforms as public (or permissionless), in which anybody can join and participate in the network consensus, or permissioned, in which consensus is achieved by a set of known and identifiable peers [22]. Bitcoin [23] and Ethereum [24] are examples of public blockchains. Permissioned blockchains are particularly interesting in business applications in which the parties need to identify each other [22,25]. Furthermore, permissioned blockchain consensus protocols usually expend less computational resources and can reach better transaction latency and throughput [26]. In their survey about blockchain architectures, Ismail and Materwala [27] also discussed the difference between Single-Ledger-based (SL) and Multi-Ledger-based (ML) architectures. SL architectures support both public and permissioned blockchain networks. They constitute most implementations, covering the three first blockchain evolution tiers: currency, smart contracts, and decentralized applications. The ML architecture concept came originally from Hyperledger Fabric [25]. It was the first platform to enable confidential and private transactions among distinct peer subgroups, introducing a protection level between pieces of information with different access privileges.

### 2.3. Blockchain-Based Pki Application

In this paper, we also develop a blockchain-based PKI application to test our inter-NMI network. This section explains the elementary concepts about the importance of this kind of application to LM and why blockchain can be a promising platform to implement it.

#### 2.3.1. Digital Signatures and Smart Meters

The digital signature is one of the main applications of public-key cryptography [28]. Its implementation mechanism relies on a pair of asymmetric cryptographic keys and a digital certificate. The digital certificate is the attestation made by a trusted third party that the public key belongs to the sender. The sender calculates the information cryptographic hash (digest) and encrypts it using his/her private key. In a complementary manner, any entity can verify the digital signature using the sender’s public key in the digital certificate to decrypt the digest and check its correspondence to the original piece of information. This process attests to the information’s integrity, authenticity, and non-repudiation.

Digital signatures can be a powerful tool to protect the smart meters’ LR chain against fraud and security attacks. This conjecture comes from the premise that a smart meter can store and protect a pair of asymmetric cryptographic keys. A meter can sign its measurements, raw data, or any LR information, providing evidence of integrity and authenticity. Furthermore, cryptographic directives can enable more sophisticated security mechanisms such as cryptography token-based access control and software integrity verification and updates. However, digital signatures and certificates are little explored concepts in the LM scope. The literature reports a few cases involving the use of public-key signatures to ensure the authenticity and integrity of measurements and to control LR software updates [29]. We can cite examples related to the protection of the LR information in electronic speed meters [30] and in the verification of sensing data from sphygmomanometers [31]. To the best of our knowledge, no work reports practical results on the use of digital certificates in measuring instruments.

#### 2.3.2. Public-Key Infrastructure

Systems implementing digital certificates usually require a Public-Key Infrastructure (PKI). A traditional PKI demands some entities performing specific roles. The main one is the Certification Authority (CA). The CA is responsible for emission, distribution, renewal, revocation, and digital certificates’ management. In practice, the CA signs the digital certificates using its private key, attesting to their correspondence to the respective entities. The Root-Certification Authority (Root-CA) is the first CA in the certification chain. The Root-CA is responsible for verifying and auditing the other CAs. Furthermore, it is responsible for the emission, distribution, renewal, revocation, and management of the CAs’ digital certificates. Finally, we have the Registration Authority (RA), which provides the interface between the CA and the certificate owner (i.e., the entity that acquires the digital certificate). The RA receives, validates, and forwards requests to the CA.

#### 2.3.3. How Blockchain Can Help

Blockchain-based PKI is an alternative to CA-based PKI. In the last three years, different works have proposed this idea, especially in contexts involving IoT applications [32,33,34]. A blockchain-based PKI contraposes a conventional CA-based PKI because it eliminates the dependency on a Trusted Third Party (TTP). Consequently, a blockchain-based PKI does not depend on CAs to sign digital certificates. Digital certificates become blockchain digital assets that link an entity to a public key. Once the blockchain’s intrinsic properties ensure the ledger’s integrity and immutability, all the involved entities can trust each of the stored digital assets (i.e., certificates).

In the context of LM, a blockchain-based PKI can significantly save costs, besides reducing the dependency on a TTP. Usually, smart meters are very inexpensive devices, and any minimal expenses related to issuing a CA-based digital certificate can be prohibitive. Furthermore, TTP dependency can be challenging, especially when measurement frauds are recurrent and very profitable (e.g., the trade of measured goods in developing countries), encouraging malicious entities to collude and bribe the TTP. A blockchain-based PKI can provide a tradeoff between security features and costs. Different participants interested in ensuring the reliability of measurements from smart meters (i.e., manufacturers, industry, vendors, notified bodies, government, consumers’ representatives) can constitute a consortium and implement a blockchain. Each participant provides a small set of peers and takes part in the blockchain consensus. The blockchain uses a smart contract to collect each meter’s public key at manufacturing time and store it in the ledger. Since the ledger is immutable, it permanently links the public key to its respective owner (i.e., smart meter). A second smart contract can implement digital signature checking services, enabling any entity with access to the blockchain to execute this task without the need for a TTP.

Peters et al. [14] described how a blockchain-based PKI would work in the context of a project like the EMC. Inner nodes (i.e., peers that integrate the blockchain network) can add and revoke digital certificates. These nodes can belong to manufacturers, notified bodies, and market surveillance entities, for instance. Contrary to those, outer nodes (i.e., entities that only mirror the blockchain and do not write directly to it) are individual measuring instruments and users of these devices. They can request the checking of signed assets to be confirmed by the inner nodes.

## 3. The Inter-Nmi Blockchain Network Architecture

The suitability of any blockchain-based measurement application depends on the existence of a network (or consortium) composed of entities interested in these applications’ correct functioning. It does not make sense to talk about a blockchain made up of a single organization. A blockchain network needs a set of peers (i.e., machines) provided by the different organizations that integrate it. These organizations must also be independent, with complete freedom and responsibility in managing their respective peers. The measurement applications deal with sensitive information (e.g., metering information can reveal a consumer’s habits and violate his/her privacy). Thus, these applications rely on access control mechanisms that determine which peers can access specific information in the ledger. These aspects indicate that we need a decentralized (i.e., organizations manage their peers independently) and permissioned (i.e., organizations identify and authenticate each of their peers) blockchain network, as also indicated by Thiel and Wetzlich [12]. Moreover, managing which entities can get information and invoke blockchain applications (i.e., smart contracts) can demand different access levels. Therefore, we also propose the adoption of a multi-ledger-based architecture, embedding access privileges in different ledger instances.

### 3.1. Properties of a Decentralized Blockchain Network

**Figure 1:**
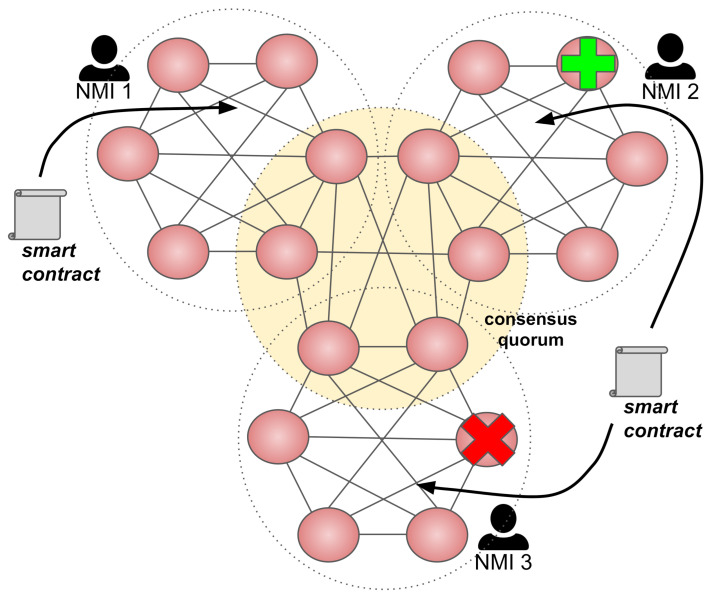
An inter-National Metrology Institute (NMI) blockchain network with a decentralized and permissioned architecture.

The idea of a decentralized network consists of the fact that each participating organization is responsible for managing, controlling, and maintaining its peers. Figure 1 illustrates this scenario, describing a blockchain network with three independent organizations (in our particular case, NMIs).A quorum formed by peers from each organization is responsible for getting consensus. One can observe that organizations are entirely free to add and remove peers (as the “plus” and “x” icons illustrate) without the need for centralized coordination.Likewise, organizations can add and remove applications, which are represented by smart contracts. Organizations can also share smart contracts, which means they provide the same application.

An organization needs to observe a set of steps upon joining the blockchain network. These steps help to understand the implications related to the decentralized administration strategy. They are the following:

1. Affiliation: This occurs when a particular organization joins the blockchain network. Such a decision can occur voluntarily (e.g., the organization wants to contribute to an application of interest) or through formal agreements (e.g., several organizations establish a contract to maintain a particular application);
2. Availability of peers: The organization contributes a specific number of peers. A peer is usually a machine (virtual or physical) that participates in the blockchain network, either as a data replicator (i.e., the peer stores and propagates the blocks) or as a member of the consensus quorum. The number of peers that each organization contributes depends on the format of its membership. Organizations that integrate the network through formal agreements must keep a specific number of peers, also specified in the contract;
3. Peers’ identification: Each organization is responsible for identifying and authenticating its peers. At the same time, each organization must broadcast its peers’ identities to the other organizations. We describe this step in detail in the next subsection;
4. Peers’ maintenance: Each organization is responsible for maintaining its respective peers. Thus, whenever a certain peer becomes inoperative, the organization is responsible for its repair or replacement, repeating the other necessary steps. If the affiliation is voluntary, the organization is completely free to remove or add new peers whenever it seems necessary. We state that peers’ maintenance is a continuous and important activity to the blockchain network availability.
5. Peers’ removal or revocation: Each organization is responsible for notifying the others whenever it permanently removes a peer from the network, or when the peers’ credentials are no longer valid (i.e., expiration or some security compromise).

Although the activities associated with the described steps are very similar to ordinary network administration activities, the first ones are more straightforward. The reason is that features inherent to blockchain technology (i.e., replication, consensus) encapsulate some of a blockchain network’s most critical tasks. For instance, a blockchain network does not need backup mechanisms since the correct ledger replication among the network’s peers already guarantees its recovery. Whenever an organization adds a new peer or an active peer remains offline for some time, these peers will restore the ledger’s current state as soon as they connect to the others.

### 3.2. Properties of a Permissioned Blockchain Network

A permissioned blockchain implies the need for identifying any peer that integrates the network. Besides, if the blockchain network deals with sensitive information, privacy can be a concern, and the identification mechanism can help implement the ledger’s access control. Regarding the peers’ identification, the most appropriate solution consists of using protocols based on public-key cryptography. The majority of blockchain implementations already standardize this strategy. Each peer must have a key pair (public and private) necessary to sign its transactions. In turn, each organization is responsible for disclosing its peers’ public keys to other organizations. An interesting aspect is the following. Since our initiative proposes a decentralized network, each organization is independent in managing the assignment of its peers’ cryptographic keys. For example, an organization can opt for a PKI to assign and verify keys and eventually use it to disclose its peers’ public keys to other organizations. Within the same concept, another organization may choose to manage the key pairs centrally, using a specific system (e.g., a Hardware Security Module (HSM)) to store and distribute its peers’ keys securely. In practice, each organization is can choose a model whose security is more appropriate for its applications.

### 3.3. Advantages of Using Multiple Ledgers

In a permissioned blockchain network, peers use their credentials (usually a pair of cryptographic keys) to access the ledger. However, this mechanism can pose one more challenge. Since the ledger data structure consists of blocks, the peers need access to their entire content to replicate them. Therefore, it can be difficult to restrict access to their specific parts of the blocks (i.e., transactions). A strategy to avoid this problem is the adoption of multi-ledger-based blockchain architectures.

In a multi-ledger-based architecture, the blockchain network manages different chains of blocks. Certain chains are accessible to a specific set of peers, while others are not. This strategy is efficient. However, it requires proper application planning. It is necessary to separate information that requires specific secrecy or access policies in different chains.

An additional strategy to deal with the same problem is data encryption. The applications encrypt any sensitive information before inserting it into the ledger. This approach implies a computational overhead and can even make the direct computation of data using smart contracts difficult. An alternative is to employ homomorphic encryption strategies that enable computation on the cryptographic domain. The works of Peters et al. [15] and Yurchenko et al. [16] already discussed this idea’s suitability in applications involving blockchain and smart meters’ data privacy. Furthermore, the blockchain network can also combine a multi-ledger-based architecture and data encryption. This strategy can be straightforward in providing access control and confidentiality, addressing privacy concerns in two security tiers.

## 4. Case Study: A Blockchain-Based Pki for Smart Meters

### 4.1. How a Ca-Based Pki Works

We start by discussing how one can implement a CA-based PKI for smart meters. The understanding of the advantages and drawbacks of a CA-based PKI model is the first step to conceive of a blockchain-based PKI model. The CA-based PKI model described here comes from the work of Melo et al. [5]. The authors presented an ongoing project proposing an architecture to implement digital certificates in fuel dispensers. The main objective was to reduce and prevent frauds that tamper with fuel measurements, which is a very disseminated practice in Brazil [18]. Rodrigues Filho et al. [35] reported that, in Brazil, frauds related to fuel dispensers result in economic losses in the order of USD 300 million per year for all society. These reasons motivated the Brazilian National Institute of Metrology, Quality, and Technology (Inmetro (https://www.inmetro.gov.br (accessed on 30 December 2020))) to regulate and enforce the insertion of digital certificates into fuel dispensers [5].

**Figure 2:**
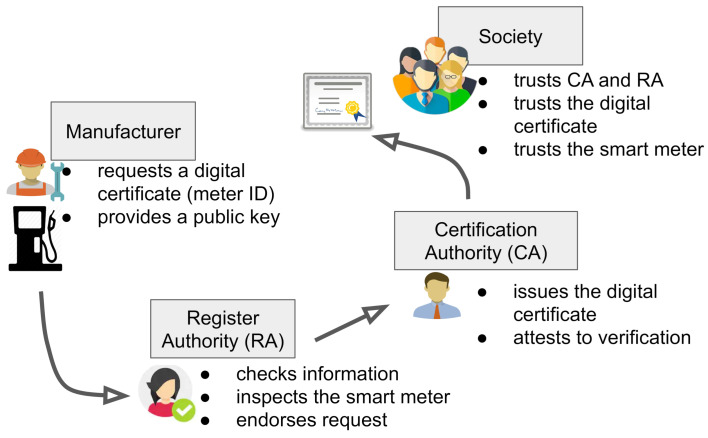
CA-based PKI scheme describing the RA and CA roles.

We depict the Inmetro’s PKI model in Figure 2. The scheme shows how PKI authorities interact to provide digital certificates for a smart meter. This process is detailed in the following consecutive phases.

#### 4.1.1. Smart Meter Manufacturing

The smart meter project must contemplate the insertion of a hardware-based cryptographic module. This kind of module usually includes functions for key generating and secure storage. Due to its critical features, the cryptographic module must be sealed in the meter at manufacturing time. As a security premise, the cryptographic module must be a tamper-proof component. As a consequence, any attempt at removing the cryptographic module from the meter must cause permanent damage to its components, destroying any previously-stored cryptographic key. The cryptographic module can sign any LR information generated by the meter. Furthermore, the secure storage protects the private key secrecy (i.e., the private key never leaves the cryptographic module) and can keep any required digital certificate. The cryptographic module also needs to deliver an interface that exports the public key and enables some mechanism to prove that it holds the respective private key.

#### 4.1.2. The Initial Verification

A “metrological” RA is responsible for the initial verification of each new meter. In this task, the RA has a double function. Firstly, it checks if the meter presents the expected project features and satisfies a set of functional requirements, as well as proceeds with metrological tests to assure that the meter is providing the correct measurements. After that, the RA verifies the asymmetric cryptographic key pair, by attesting that the meter stores its private key securely and also that the meter can export its public key. We emphasize that the RA does not access the meter’s private key. The RA only checks the correspondence between the public and private keys by using some challenge-response protocol. Finally, the RA sends the meter information (including its public key) to the CA responsible for issuing the digital certificate.

#### 4.1.3. Issuing the Digital Certificate

After receiving the meter information validated by the RA, the CA can proceed with issuing the digital certificate. The digital certificate associates the meter with its public key in a manner that is unique and irrefutable. Thus, one can trace any signed measurement to the respective meter. The information has verifiable integrity and authenticity, and no entity can deny its source. The CA issues the digital certificate and sends it to the manufacturer. The manufacturer is responsible for embedding the certificate into the respective meter.

#### 4.1.4. The Meter’s Deployment

Once the meter has its digital certificate properly embedded, the manufacturer is authorized to sell it to an owner. We can say that the owner deploys the meter, which can involve different scenarios according to the respective meter’s use cases. These use cases also determine the need for future inspections that demonstrate if the meter is working as expected. Inspections can include the verification of the digital signatures of measurements and LR information. Furthermore, different smart meters can provide these digital signatures in a manner that enables their verification directly by the meter’s final user. For instance, a fuel dispenser can print a piece of paper with the LR information (e.g., a transaction ID, a meter ID, the timestamp, and the traded fuel amount) of a commercial transaction, together with the respective digital signature. After that, the driver (who is the final user here) can verify this digital signature by using a service from the CA. A correct digital signature evidences the integrity, authenticity, and non-repudiation of the LR information, assuring that the data did not suffer any tampering after leaving the meter.

### 4.2. Conceiving of a Blockchain-Based Pki for Smart Meters

In contrast with the CA-based PKI model described in Section 4.1, we present a blockchain-based PKI. This new architecture simplifies the maintaining of digital certificates (creation, verification, revocation) and also reduces the dependency on TTPs.

**Figure 3:**
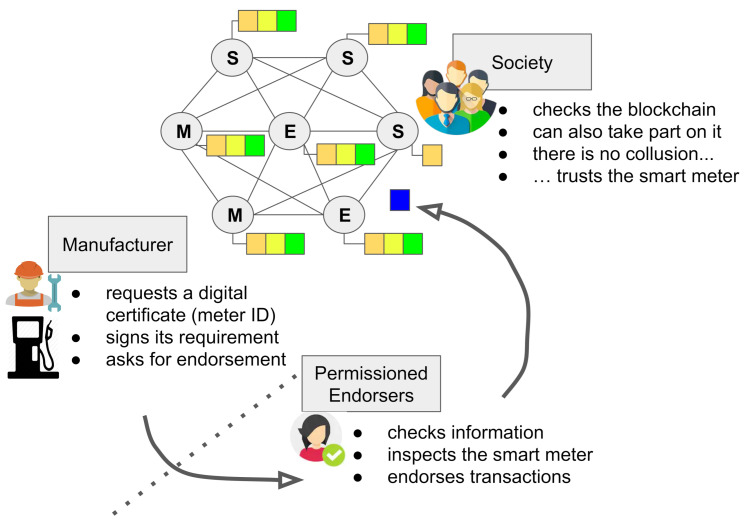
A blockchain-based PKI for smart meters scheme. S, Society; M, Manufacturer; E, Endorser.

Figure 3 illustrates how the blockchain-based PKI works. First of all, we assume the existence of a consortium among several independent organizations that provide peers (i.e., computers) to integrate the blockchain network. These organizations can represent the interest of Manufacturers (Ms), entities from Society (S), and institutions responsible for assuring the correct behavior of smart meters and endorsing this (Endorsers (Es)). We call this last group Permissioned Endorsers (PEs). Notice that the mentioned classes of organizations are only an illustrative example. We can have many more entities taking part in the blockchain and representing different objectives.

The peers in the blockchain network keep a distributed ledger that stores the meters’ public keys. Every data entry in the ledger constitutes an association between a meter and its respective public key. Thus, we can say that the blockchain digital assets replace digital certificates. Furthermore, the blockchain peers replicate the ledger among each other, in a manner that any peer can verify whether a public key belongs to a specific meter. By doing this, the blockchain network replaces the CA role. This is a direct consequence of using blockchain: we reduce (and in some cases, we even eliminate) the need for a TTP.

For a better understanding, we describe the blockchain-based PKI workflow in four phases, just like we did with the CA-based PKI. Although phases in both models are equivalent in terms of features, they present distinct processes and complexity.

#### 4.2.1. Smart Meter Manufacturing

The meter manufacturing phase in the blockchain-based PKI is practically the same as described in Section 4.1. The manufacturer needs to embed into its meter a secure tamper-proof cryptographic module that generates, stores, and protects the asymmetric cryptographic key pair. However, the digital certificate becomes a data entry in the blockchain. Therefore, the manufacturer needs to ask for initial verification and an endorsement for the meter’s public key.

#### 4.2.2. The Initial Verification

The initial verification is also similar to the corresponding phase in the CA-based PKI model. We still need a “metrological” RA role that must do the smart meter’s metrological inspection and the cryptographic key pair verification. The main difference is that the PE does not require asking for the digital certificate issuing. After proceeding with all the verification steps, the PE submits a transaction to the blockchain that inserts the meter’s public key into the ledger.

A challenge here is to limit the possibility of the PE being a malicious entity that forges public keys. For instance, a malicious PE could associate a smart meter with a public key whose private key is not stored in the meter’s cryptographic module. Firstly, one must consider that, since the PE is the only entity authorized to submit transactions to the blockchain, it should be a reliable entity. Indeed, the CA-based PKI depends on the same assumption for RAs. Therefore, we argue that a malicious PE can only succeed with the cooperation of the manufacturer. This condition also implies that the PE and the manufacturer need to agree about fraud. The blockchain-based PKI enables an additional countermeasure in this case. We can establish security policies requiring that more than one independent PE proceeds with the initial inspection. This strategy makes collusion attacks more expensive and, consequently, more difficult. Due to this possibility, we can reinforce the argument that blockchain helps to reduce the dependency on TTPs.

#### 4.2.3. The Digital Certificate Issuance

As we mentioned before, the blockchain-based PKI eliminates the need for digital certificates’ issuance. The attestation that a public key belongs to a specific meter comes directly from the blockchain. If the blockchain has a data entry with the meter’s public key, then all the involved stakeholders trust this information.

#### 4.2.4. The Meter Deployment

In the blockchain-based PKI model, the meter deployment depends on the existence of a blockchain data entry that associates the meter ID with its public key. The blockchain becomes a database that indicates whether a manufacturer can sell a meter. More than that, a future owner can verify the meter register in the blockchain before concluding the acquisition. Once the meter has an owner, its use case follows the regulation associated with the meter’s use cases. In this aspect, there is no difference between the two PKI models. However, the verification of a digital signature is distinct: the blockchain can provide this service as a smart contract. In practice, any entity interested in verifying a digital signature can invoke a smart contract indicating the meter’s ID, the LR information, and the signature digest. Any peer in the blockchain network can execute the smart contract, checking whether the meter ID has a data entry on the ledger and confirming whether the signature digest corresponds to the stored public key. Furthermore, the blockchain logs any signature verification in the ledger. This register constitutes a thorough audit tool.

## 5. Implementation Using Hyperledger Fabric

In this section, we present a practical implementation of our proposal. In fact, we build an inter-NMI blockchain network that connects remote servers in Germany and Brazil. We test this network by implementing a blockchain-based PKI application that permanently stores smart meters’ public keys and provides digital signature checking services. The details about this implementation, its setup, performance, and results are described in the following subsections.

### 5.1. Overview of Hyperledger Fabric

We implement our inter-NMI blockchain network and the blockchain-based PKI application using Hyperledger Fabric (Fabric) [25]. Fabric is a complete platform for implementing a permissioned blockchain able to deal with more than 3500 transactions per second (tps) [25]. We utilized the following key concepts and components from Fabric for our inter NMI blockchain network:

1. Smart contract and chaincode: In Fabric, a smart contract, which is also called a chaincode, implements the executable logic of our PKI implementation.
2. Peer: We utilized thee stages of the peer (endorsing, committing, and anchor peer) from the Fabric component. In our implementation, peer0 (we have named 2 peers as peer0 and peer1 for each NMI) from PTB and Inmetro works as the endorser (to endorse the transaction), committer (commits the block received by orderer service), and anchor peers (communicates between two different NMI organizations) at a time.
3. Ordering service: The ordering service is responsible for creating consensus, receiving endorsed transactions from clients, and ordering the replicated transaction blocks to peers [15]. In our implementation, we used the “solo (single node ordering service)” and the “Raft [36] (Crash Fault-Tolerant (CFT) ordering service using distributed consensus)” ordering service for the single-host and multi-host deployments, respectively.
4. Membership Service Provider (MSP): To maintain the identities of all NMI nodes in the network, the MSP component is used for our inter-NMI blockchain network. The MSP component from Fabric issues the crypto resources for authentication and authorization for each NMI organization.
5. Channel: In Fabric, a channel represents the private tool for communicating between several network members for private and confidential exchanges. Both NMIs (PTB and Inmetro) communicate through a “nmichannel” in the NMI blockchain network.

We utilized the above-mentioned components from Hyperledger Fabric to realize our proposed scheme from Figure 3 in the following steps:

- Manufacturer (M): Meter manufacturing is similar to CA-based PKI where the manufacturer needs to provide the secured tamper-proof cryptographic module. In our proposal, we denote the digital certificate as a data entry phase in the blockchain. For simplicity, we created a client using the Python SDK (https://github.com/hyperledger/fabric-sdk-py (accessed on 30 December 2020)) of Fabric to generate the asymmetric cryptographic key pair using the Elliptic Curve Digital Signature Algorithm (ECDSA). In this phase, the manufacturer requires initial verification and endorsement for the public key of the smart meter from the PE.
- Permissioned Endorsers (PEs): Being a trusted entity only, the PE is responsible for metrological inspection of smart meters and can submit transactions to the blockchain. The transaction represents inserting the public key of the smart meters into the ledger. To represent PE in our implementation, we used the “peer” concept from Fabric.
- Smart contract: The verification of the digital signature is implemented in a smart contract that is served by the blockchain. In Hyperledger Fabric, a smart contract is a chaincode that executes our logical operation.
- Entities from Society (S): Any authorized entity from society interested in digital signature verification can invoke a smart contract by providing the meter ID, LR information, and signature digest. We created a client application for the verification of this step and to interact with the smart contract.

### 5.2. Single-Host Deployment

In this experiment, one organization exists that provides the PEs that are responsible for verifying the smart meters (as described in Section 4.1) and that can generate digital assets linking a meter to its public key. We created a smart contract that encapsulates all the features related to the insertion of digital assets into the ledger and the verification of digital signatures. The source code and the description of its functional aspects are available in a public repository on GitHub (https://github.com/wsmelojr/blockmeter (accessed on 30 December 2020)).

We used a Python program to simulate the smart meters and PEs. Each smart meter instance has an asymmetric cryptographic key pair. The meter also knows how to export its public key and how to generate signed measurement information. PE instances have the permission to write digital assets into the ledger. Every time we instantiate a client meter, a PE process requests its public key and submits a transaction to the blockchain. All the peers in the blockchain network replicate and validate the transaction by consensus. If the information is consistent, the peers append a new digital asset to the blockchain that links the meter with its respective public key. Once the meter’s public key is in the ledger, anyone with access to the smart contract can invoke it, to verify the digital signature of the LR information [17].

### 5.3. Multi-Host Deployment

In our previous setup in Section 5.2, the Hyperledger Fabric components were deployed as Docker containers on a local server. For demo purposes, it was sufficient to deploy smart contract and other containers in just one host. However, for practical uses on many servers, the initial network described in Section 5.2 needs to distribute the Docker containers to other NMI servers. The Hyperledger Fabric framework does not have any official documentation using any external network with a multi-host setup. The configuration files using a containerization system running in a single server do not directly work with a multi-host deployment. Still, in our current setup, we wanted to deploy Fabric in a multi-host network using the PTB server in Germany (PTB-S1) and the Inmetro server (Inmetro-S2) in Brazil. The server configuration is described in Table 1.

There are multiple ways to deploy Fabric in a multi-host environment. We tested the two following ways for a multi-host deployment:

- Configuring communication between peers (without the orchestration tool)
- Docker swarm-based orchestration

#### 5.3.1. Configuring the Communication between Peers (Without the Orchestration Tool)

In a multi-host network for PTB and Inmetro, Docker containers can communicate with each other by deploying specific containers according to their host IP address. By changing the configuration files and specifying the network as an external network, we were able to implement such a network. For this setup, there is no dependency on other external components. As we intend to add more NMIs in the future, this approach leads to configuring the networks dynamically being a bit challenging. During the implementation, we also realized that initially, for demonstration purposes, we needed to provide some configuration tool that could be managed by the clustering approach.

#### 5.3.2. Docker Swarm-Based Orchestration

One of the challenges in our implementation was to build a multi-host deployment and establish the communication between the Docker containers in different networks. At the beginning of the setup, points like managing, scaling, deploying, restarting, reproducing, updating, and deleting of the Docker containers during their entire life cycle were major concerns. Especially from the security point, it was not clear how it might be certain that the containers are running on the particular host machine on which we intended them to run. After a container is created, it is not possible to make changes to its configuration other than renaming it. Another requirement for the containerized application across the multiple nodes in our setup is to be scalable and conveniently portable. For this, we utilized a Docker swarm (https://docs.Docker.com/engine/swarm/ (accessed on 30 December 2020))-based orchestration provided by Docker Desktop.

Docker swarm creates a cluster of nodes by using the networking driver called “overlay” to establish the communication between the containers across multiple nodes. This creates the swarm-wide bridge network where the containers across hosts on the same virtual network can access each other as if they were running in a Virtual Local Area Network (VLAN). In a swarm network, nodes are categorized as “managers/leaders” (control the network, balance the load, and responsible for the container recovery if needed) and “workers” (help with running the containers as per service deployment).

In our setup, we utilized both PTB-S1 and Inmetro-S2 servers as “manager/leader” nodes. By using the “Docker service”, containers are deployed and orchestrated across these two servers. By default, Docker randomly pushes the containers to the nodes of the network. However, in the NMI blockchain network case, we mapped each server to a specific node that was owned by the corresponding NMI. We just needed to open some ports between the servers for communicating through the “overlay” network. One of the main advantages is that we did not need to expose all the peer and orderer ports in between the server. Most of the Fabric components can be used in the internal network of the NMI. In the current setup, our NMI network consists of two NMIs (PTB and Inmetro) using the following components from Hyperledger Fabric:

- One channel (nmichannel)
- Two peers per NMI (peer0, peer1)
- Five orderers (using the Raft protocol)
- One smart contract (PKI-based)
- Membership Service Provider (MSP) for crypto materials

In this experiment, nodes were hosted in two different servers and networks. Each server was running Ubuntu 18.04 LTS and Docker Version 19.03.14. For this particular setup, we used Hyperledger Fabric Version 1.4.9 as the Raft orderer service, which was introduced in Fabric Version 1.4.1. Each NMI organization had two peers (peer0 and peer1). Peer0 was the endorser peer for both NMIs. The communication between the two NMIs was through an “nmichannel” and used the Transport Layer Security (TLS) protocol.

In the Docker swarm, the overlay network driver creates the distributed network between the Docker hosts. The host specific network allows the secure communication between the containers. We initialized the Docker swarm node in PTB-S1 and activated it as a “manager/leader” node. The Inmetro-S2 host joins the network as a “manager/leader” using the generated token from PTB-S1. The Docker swarm adds the overlay network “nmiNetwork” between both servers. All nodes can now communicate through this network. For simplicity, we used the cryptogen tool from Fabric to generate the crypto resources. All the nodes in the “overlay” network need to share the same crypto resources. We generated crypto materials in PTB-S1 and distributed them also into Inmetro-S2. The peers (peer0.ptb and peer0.inmetro) of PTB and Inmetro servers communicate using the gossip protocol (messages are encrypted with Transport Layer Security) of Fabric. The gossip protocol basically ensures the synchronization between the peers and checks if the same ledger is maintained everywhere.

**Figure 4:**
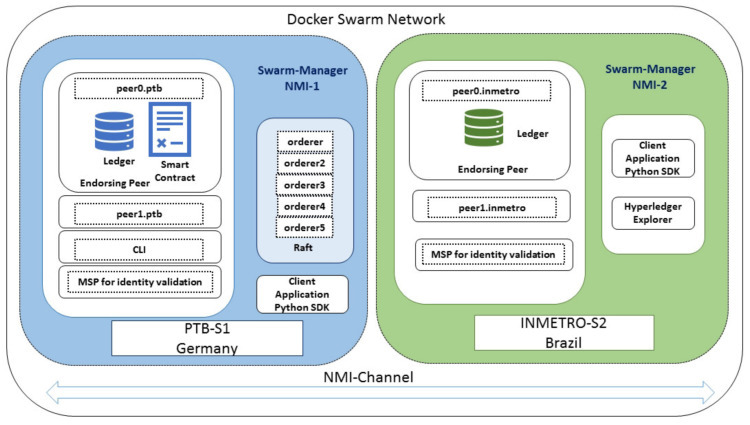
Docker swarm-based inter-NMI blockchain network. MSP, Membership Service Provider.

After that, we deployed the Docker services based on “raft” by running containers in each host described in Table 2 and Figure 4. In our experiment, peer0.ptb and peer0.inmetro also act as anchor peers, which can be discovered by other peers (peer1.ptb and peer1.inmetro) in nmichannel. The command line tool CLI container is hosted on PTB-S1. Therefore, creating the genesis block and the channel is initiated in PTB-S1 for now. All the peers from PTB and Inmetro can join this channel through the Docker swarm network. In the next step, we also installed the chaincode in both NMI nodes. However, in Fabric, chaincode “instantiation” can be done only on one peer, which is deployed in peer0.ptb peer for now. However, in the future, this will be also possible from the Inmetro-S2 server, if required.

### 5.4. Consensus Using the Raft Protocol

In our experiment in Section 5.2, we used the “solo” orderer service from Fabric, which is good for development. While implementing the NMI blockchain, we realized that there was a need for a protocol for implementing distributed consensus. Normally, for single instance databases, the value is taken and stored in one server. When database servers are in a Docker swarm cluster, the concern is to correctly achieve distributed consensus. Fault tolerance mechanisms also play a major role as there could be a strong chance of node and network failure. As we were already using a permission-based system, protection against malicious nodes was not considered here.

Raft [36] is a distributed “Crash Fault Tolerant” (CFT) protocol, which manages the replication of the log and based on an implementation of the Raft protocol in “etcd” from Fabric. This consensus algorithm also tackles two other issues: leader election and safety. In our experiment, we had five orderer nodes in a channel. According to the Raft protocol, the loss of two nodes can be tolerable. For the simplicity of the setup, we ran five orderers in the PTB-S1 server. Typically, a node can be in three states: leader, follower, and candidate. Initially, all five orderer nodes start with a pseudo-timeout known also as a “heartbeat” and self-promote the candidate states. If a candidate receives a quorum of votes, it will be promoted as a leader node [36]. Once the leader is elected, it can process the request from the client.

Although in the NMI blockchain network, we ran our experiment just for two NMI organizations, we are confident that in the future, other NMI organizations can also join the network by using the Raft consensus mechanism. In Fabric, each channel runs on a separate instance of the Raft protocol. Several NMI organizations can communicate with different channels, and each instance from each channel can elect a different leader. One more advantage of using a Raft-based orderer service is that each NMI can have its own orderer node hosted, which certainly achieves the benefits of decentralization. Each NMI organization can be treated equally as each node will have an equal probability of becoming a leader. Still, we tested our concept on a blockchain network for limited NMIs. Hence, we need to consider the fact that Raft is limited in scalability due to its architecture [37]. At this moment, for our use case, improving the “lack of fault tolerance” is more important than the “lack of scalability”.

## 6. Results and Discussion

Within this article, we experimented initially with the single-host setup using the solo orderer service. Then, we fulfilled the requirement by adding servers to the system that represents different NMIs in the metrology field. We divided our multi-host setup into two categories. One was to connect the servers by establishing the communication between peers without the use of any external component or orchestration tool. The other was to add peers using the orchestration tool named Docker swarm. In both cases, we tried to figure out the impact on the transactions and the No. of blocks using the solo and Raft orderer service, which we present in the following subsections.

### 6.1. Overview of the Hyperledger Explorer Tool

**Figure 5:**
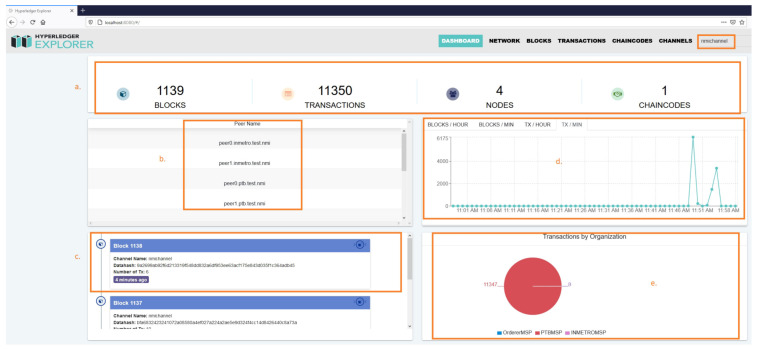
Results of 64 clients/instances in swarm-based network signature verification.

To monitor the blockchain information and our experiment, we used the container-based distribution of the Hyperledger Explorer (https://www.hyperledger.org/use/explorer (accessed on 30 December 2020)) tool. This user-friendly visualization tool helps one see the network information, the number, and the details of blocks and transactions. Individual valid and invalid transaction details and relevant information stored in the ledger can also be seen from the reports, which gradually increase, depending on the test setup. From Figure 5 marked as Section (a–e), the visualization tool Hyperledger Explorer helps us to analyze the following:

- Counters: Section (a) from Figure 5 represents the No. of blocks, transactions, nodes, and the chaincode.
- List of peers: Section (b) represents the peer names of PTB and Inmetro along with five orderers in the NMI network.
- Block notification: Section (c) represents the block that is lastly added and contains the channel name (the block was created through “nmichannel” and provides privacy to the network), the data hash (contains the encrypted code), and the number of TX (describes the number of transactions per block) fields.
- Metrics: Section (d) represents the statistics of the blocks and transactions per channel. The graph also shows the metrics per block or transaction per hour/minute.
- Transactions: Section (e) represents the transactions by organization. For simplicity, we generated crypto resources on PTB-S1 and mapped “PTBMSP” in the Explorer configuration file.

### 6.2. Using Hyperledger Explorer Tool for a Single Host

To evaluate our implementation, we experimented on a server running Hyperledger Fabric 1.4 LTS (https://www.hyperledger.org/use/fabric (accessed on 30 December 2020)). The hardware consisted of an Intel Server S2600CW with 256 GB RAM and two Xeon E5-2650 v4 CPUs running at 2.2 GHz. This server hosts all the blockchain network peers, which are comprised of Docker containers and the concurrent client instances [15]. In Hyperledger Fabric, the chaincode is equivalent to a smart contract as it handles the business logic [17]. We deployed the blockchain network for our test setup using just one organization with one peer fabric infrastructure, which works as both the endorser and committer. In this particular scenario, the number of organizations does not affect the number of transactions submitted. For the client application, we used the Python3 modules multiprocessing package, which enables concurrent processes and threads.

To replace the need for the digital certificate, we used the Elliptic Curve Digital Signature Algorithm (ECDSA) asymmetric keys, which create the digital assets. By using the Python ECDSA library (which implements our cryptosystem) and the Fabric Python SDK (https://github.com/hyperledger/fabric-sdk-py (accessed on 30 December 2020)), the client application invokes and queries the chaincode services provided by the blockchain network.

To conduct the test, we initially started the blockchain network with an empty ledger. We tested up to 64 client instances by defining the ledger dataset of 100 unique meter IDs for each client instance. The test setup concurrently starts all the concurrent client instances and runs over an individual thread. The client application sends the transaction proposals to the peer, which is basically invoking the chaincode. The peer endorses and simulates each of the concurrent transactions by executing the chaincode. Meanwhile, the peer also verifies the transaction proposal, signature, and authorization of the client instances.

A peer then returns an endorsement package to the respective client instances. The blockchain network is configured with one solo ordering service with a solo consensus mechanism for ordering the transactions. Client instances broadcast the transactions to the ordering service, which then creates the blocks. The blocks of delivered transactions are also labeled as “valid” or “invalid”.

According to our client application, the Registration Authority (RA) verifies the new Measuring Instrument (MI) and confirms the correspondence between the measuring instrument’s public and private keys. The RA inserts the public key associated with its respective measuring instrument ID into the ledger. This procedure is denoted as “register meter-single host” in Table 3. We repeated this procedure at the beginning of each test round. To check the growing number of blocks and valid transactions for 4, 16, 36, and 64 clients, we added invoked chaincode results for “register meter-single host” in Table 3.

**Figure 6:**
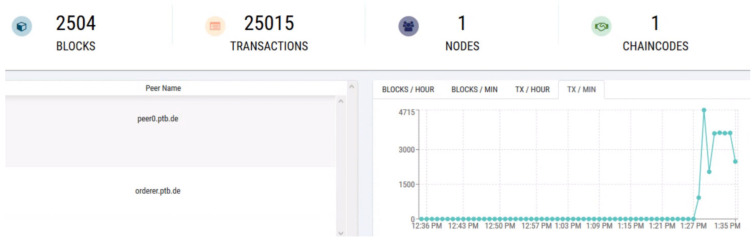
Results of 64 clients/instances for verifying the signature in a single host.

On the other hand, any interested party, here named the client, can audit the blockchain entries and ask for the digital signature checking. In this case, the client invokes the measuring instrument ID, an information piece (usually a legally relevant register), and its digital signature using its private key. The chaincode retrieves the measuring instrument’s public key and validates the digital signature by returning “true” for a valid and “false” for an invalid signature. This procedure is denoted as “signature checking-single host” in Table 4, which is inserted after “register meter” for each test round. To check the growing number of blocks and valid transactions for 4, 16, 36, and 64 clients, we added the invoked chaincode results for “Signature Checking-Single Host” in Table 4. Figure 6 shows the blocks and transaction generated numbers for 64 client instances.

### 6.3. Using Hyperledger Explorer Tool for Multiple Hosts

Similar to Section 6.2, we conducted the test in PTB-S1 for up to 64 client instances by defining the ledger dataset of 100 unique meter IDs for each client instance. It can be seen that the blockchain network has four blocks in the initial stage. Initiating the network, we created the genesis block, which is a configuration block containing no data. We conducted the test in the following two categories for the multi-host deployment. The obtained results consist of the solo and Raft orderer service with and without the orchestration environment.

#### 6.3.1. Using the Solo Orderer Service with and without the Orchestration Tool

The experiment starts all concurrent client instances and runs over an individual thread in PTB-S1. We conducted this test in both the environment with and without the orchestration tool. In the first step, we ran the test without using any orchestration tool. The Registration Authority (RA) inserts the public key associated with its respective measuring instrument ID into the ledger. We repeated this procedure at the beginning of each test round. To check the growing number of blocks and valid transactions for 4, 16, 36, and 64 clients, we used the solo orderer service and present the invoked chaincode results in Table 5.

Similar to Section 6.2, clients can audit the blockchain entries and ask for digital signature checking. In this case, the client invokes the measuring instrument ID, an information piece (usually a legally relevant register), and its digital signature using its private key. The chaincode retrieves the measuring instrument’s public key and validates the digital signature by returning “true” for valid and “false” for invalid signatures. The results obtained are represented in Table 6, which is inserted after “meter registration” for each test round. We can observe the growing number of blocks and valid transactions for 4, 16, 36, and 64 clients from Table 6.

We repeated the experiment using the Docker swarm-based orchestration tool also. After obtaining the results, we saw that the usage of the orchestration tool did not have any major impact on the No. blocks and No. of transactions.

#### 6.3.2. Using the Raft Orderer Service with and without the Orchestration Tool

The experiment concurrently started all concurrent client instances and ran over an individual thread in PTB-S1 using the Raft orderer service. We conducted this test in both the environment with and without the orchestration tool. The Registration Authority (RA) inserts the public key associated with its respective measuring instrument ID into the ledger. We repeated this procedure at the beginning of each test round. Table 7 represents the increasing number of blocks and valid transactions for 4, 16, 36, and 64 clients using the Raft orderer service in the orchestration environment (Docker-swarm).

Similar to Section 6.2, the results obtained for signature verification are represented in Table 8, which is inserted after “meter registration” for each test round. To check the growing number of blocks and valid transactions for 4, 16, 36, and 64 clients, we added the obtained results in Table 8 in the orchestration environment. We repeated the experiment without the Docker swarm-based orchestration tool also. After obtaining the results, we saw that the usage of the orchestration tool did not have any major impact on the No. blocks and No. of transactions.

After running the experiments in both the single host and multiple hosts, we certainly found significant differences. While comparing the results for the meter registration mentioned in Table 3, Table 5 and Table 7, we saw that the No. of blocks and transactions increased only by two due to the additional peer nodes added for both PTB and Inmetro NMIs. Comparing Table 4, Table 6 and Table 8 shows that having two different servers with two peers in Table 6 and Table 8 (one endorsing peer each) reduced the No. of blocks and transactions by 45% (approximately). For a single host with only one endorsing peer, the blockchain network lowered the transaction rate to the orderer, which increased the time for the block to be filled out. On the other hand, the multi-host network using two endorsing peers in two different servers (PTB-S1 and Inmetro-S2) improved the transaction rate and decreased the time for filling out the block.

Additionally, using the Raft orderer service certainly improved the efficiency. The No. of blocks and transactions did not have an impact on the usage of the orchestration tool or not. However, orderer services had a significant impact on this result. According to Table 5 and Table 7, in “meter registration”, the No. of blocks values slightly decreased for the Raft orderer service. Similarly, from Table 6 and Table 8, it can be seen that the No. of blocks and transactions values decreased for the Raft orderer service.

### 6.4. Fabric’s Performance Issues

One of the reasons we chose Fabric is due to its performance and portability on different hardware platforms. Fabric peers execute over Docker (https://www.Docker.com (accessed on 30 December 2020)) containers. This mechanism is efficient in isolating the host hardware, and one physical hardware alone can virtualize several peers. In turn, Fabric clients can use different Software Development Kits (SDKs) to invoke smart contracts and query information in the blockchain. From the client side, a smart contract is nothing more than a remote procedure call. The peers (i.e., the endorser peers in Fabric) are responsible for executing all the smart contract computations, and the clients only send and retrieve information.

There are several studies on Fabric’s peers’ performance. We can mention Androulaki et al. [25], who described Fabric’s theoretical performance in a proper benchmarking invoking, and our previous works [9,10,15], which also implemented experiments with Fabric. In Melo et al. [9], we obtained a performance of around 380 tps and a latency lower than 1.6 s by using a Byzantine Fault Tolerance (BFT)orderer service [38] composed of four replicas. Similar performance was measured in the experiments of Peters et al. [15] and Melo et al. [10]. Since Fabric is not scalable in terms of the number of transactions that the orderer service can process, we can assume these performance rates are stable, independent of the number of peers integrating consensus. However, this performance is enough for most of the applications involving digital signature checking in measurements. Usually, a smart meter needs to submit only one transaction to provide its public key. Furthermore, the blockchain network can verify 380 digital signatures per second. One must notice that Fabric’s consensus scalability constraints do not affect the smart contract’s execution since we can add more endorsers, as demonstrated in the findings of Peters et al. [15].

One last aspect that regards Fabric’s client performance in an inter-NMI network is that many of these clients will be smart meters with limited computational resources. However, to work as a blockchain client, these devices minimally need to establish an Internet connection and implement public-key cryptography directives. Although these requirements can sound hard to implement, remember that any IoT device already needs to establish an Internet connection. Besides, hardware manufacturers already count on inexpensive hardware modules that implement public-key cryptography directives efficiently and easily could integrate them into their projects. Therefore, we argue that most modern smart sensors, meters, and measuring instruments have enough computational resources to behave as blockchain clients.

## 7. Conclusions

In this article, the outcome of our work includes a blockchain-based PKI that addresses specific aspects related to the use of digital certificates in smart metering systems. We demonstrate that these devices could significant benefit from using digital signatures as a mechanism to provide the integrity, authenticity, and non-repudiation of LR information. Complementary to this, a blockchain-based PKI reduces the dependency on TTPs, simplifies the issuing of digital certificates, and also can save costs in terms of implementation and management [17]. Besides that initially, we were able to evaluate our experiments with a minimal and simplified configuration using a single-host setup, in this article, we elaborate the experiments for multi-host setup systems by connecting different NMIs to the blockchain network. To avoid a single point of failure, we added multiple hosts to the network. By adding the peer nodes, the transaction rate (No. of transactions and blocks reduced) is actually increased, and the dependency on only one peer node is reduced. Additional endorser peers allow transactions in parallel from different NMI servers, which certainly improves the transaction rate in the blockchain network. The results from the NMI blockchain network and the underlying metrology processes lead us to analyze how to integrate other NMI organizations in this blockchain network. The current implementation and the results also indicate that other NMIs can be included easily, to test the application in a boarder way. Hence, our proposed concept can be used as a real-world application in the legal metrology field.

## Figures and Tables

**Table 1:** Server configuration. PTB, Physikalisch-Technische Bundesanstalt; S1, Server 1; Inmetro, National Institute of Metrology, Quality, and Technology.

Environment	PTB-S1	Inmetro-S2
Model	Intel(R) Xeon(R) Gold 5218 CPU @ 2.30 GHz	Intel(R) Xeon(R) CPU E5-2670 0 @ 2.60 GHz
CPU(s)	64	8
Architecture	x86_64	x86_64
Hyperledger Fabric version	1.4.9	1.4.9

**Table 2:** Distribution of Docker containers.

PTB-S1	Inmetro-S2
peer0.ptb (anchor peer)	peer0.inmetro (anchor peer)
peer1.ptb	peer1.inmetro
cli	explorer
orderer, orderer2, orderer3	explorerdb
orderer4, orderer5	

**Table 3:** Register meter: Single host.

No. of Clients	No. of Blocks	No. of Transactions
4	42	402
16	162	1602
36	362	3602
64	642	6402

**Table 4:** Signature checking-single host.

No. of Clients	No. of Blocks	No. of Transactions
4	188	1570
16	628	6257
36	1410	14,080
64	2504	25,015

**Table 5:** Meter registration without the orchestration tool: multi-host-solo orderer.

No. of Clients	No. of Blocks	No. of Transactions
4	49	404
16	184	1604
36	410	3604
64	726	6404

**Table 6:** Verification of the signature without the orchestration tool: multi-host-solo orderer.

No. of Clients	No. of Blocks	No. of Transactions
4	97	784
16	336	3115
36	748	6975
64	1326	12,402

**Table 7:** Meter registration in the swarm network: multi-host-Raft orderer.

No. of Clients	No. of Blocks	No. of Transactions
4	44	404
16	164	1604
36	364	3604
64	644	6404

**Table 8:** Verification of the signature in the swarm network: multi-host-Raft orderer.

No. of Clients	No. of Blocks	No. of Transactions
4	83	716
16	289	2846
36	644	6389
64	1139	11,350

## Data Availability

Not Applicable.
